# Continuous glucose monitoring using the FreeStyle Libre Pro iQ in presymptomatic type 1 diabetes

**DOI:** 10.1007/s00125-026-06852-w

**Published:** 2026-09-07

**Authors:** Andreas Weiss, Elisabeth Huber, Franziska Reinmüller, Annette Munzinger, Ezio Bonifacio, Anette-Gabriele Ziegler

**Affiliations:** 1 Institute of Diabetes Research, Helmholtz Munich, German Research Center for Environmental Health, Neuherberg, Germany; 2 German Center for Diabetes Research (DZD e.V.), Neuherberg, Germany; 3 Center for Regenerative Therapies Dresden, Technische Universität Dresden, Dresden, Germany; 4 Paul Langerhans Institute Dresden of the Helmholtz Munich at University Hospital Carl Gustav Carus, Faculty of Medicine, Technische Universität Dresden, Dresden, Germany; 5 Forschergruppe Diabetes, School of Medicine and Health, TUM University Hospital, Munich, Germany

**Keywords:** Continuous glucose monitoring, Early-stage type 1 diabetes, Glucose variability, Paediatrics, Presymptomatic type 1 diabetes, Risk stratification, Staging, Time in tight range, Type 1 diabetes

## Abstract

**Aims/hypothesis:**

The aim of the study was to evaluate the ability of the FreeStyle Libre Pro iQ continuous glucose monitoring (CGM) system to discriminate between presymptomatic stages of type 1 diabetes and stratify progression to stage 3 type 1 diabetes. The study also aimed to compare the performance of the FreeStyle Libre Pro iQ with that of the Dexcom G6.

**Methods:**

From December 2023 to December 2025, 50 children and adolescents without islet autoantibodies (control participants) and 89 with early-stage (stage 1 or 2) type 1 diabetes underwent CGM with the FreeStyle Libre Pro iQ. Participants with early-stage type 1 diabetes were followed up for the development of stage 3 clinical type 1 diabetes.

**Results:**

CGM data were unavailable or incomplete for 13 participants (9%), leaving 126 (91%) for analysis (73 female, median age 10.0 years [IQR 7.9–12.8]). The CGM parameters used—namely the SD, CV, time with glucose levels above 7.8 mmol/l (140 mg/dl; TA140) and 8.9 mmol/l (160 mg/dl; TA160) and time in tight range—differed significantly across participants with no early-stage, stage 1 or stage 2 type 1 diabetes, and between participants with a single dysglycaemia abnormality and those with multiple dysglycaemia abnormalities. One or more of these parameters exceeded the 97.5th percentile of control values in 10 (22%) of the 45 individuals with stage 1 type 1 diabetes and in 13 (52%) of the 25 individuals with stage 2 type 1 diabetes (*p*=0.017). Two or more of the parameters exceeded the 97.5th percentile threshold in six (13%) of the individuals with stage 1 type 1 diabetes and 11 (44%) of the individuals with stage 2 type 1 diabetes (*p*=0.0077). The 97.5th percentile threshold for TA140 (i.e. >15%) was exceeded by none of the 45 individuals with stage 1 type 1 diabetes, 10 (40%) of the 25 individuals with stage 2 type 1 diabetes and six of the eight individuals who progressed to clinical diabetes within 1 year, identifying these individuals as rapid progressors. FreeStyle Libre Pro iQ values differed markedly from those previously observed using the Dexcom G6 in a similar cohort. Using the 97.5th percentile of values of control individuals to define CGM abnormalities enabled harmonisation across systems and supported the consistent identification of high-risk individuals. With this approach, the sensitivity for identifying those who progressed to clinical diabetes within 1 year ranged from 75% to 100% for the FreeStyle Libre Pro iQ and from 50% to 100% for the Dexcom G6.

**Conclusions/interpretation:**

These findings support CGM as a promising tool for staging and risk stratification in early-stage type 1 diabetes, while highlighting the need for system-specific interpretation before use in therapeutic decision-making.

**Graphical Abstract:**

**Figure:**
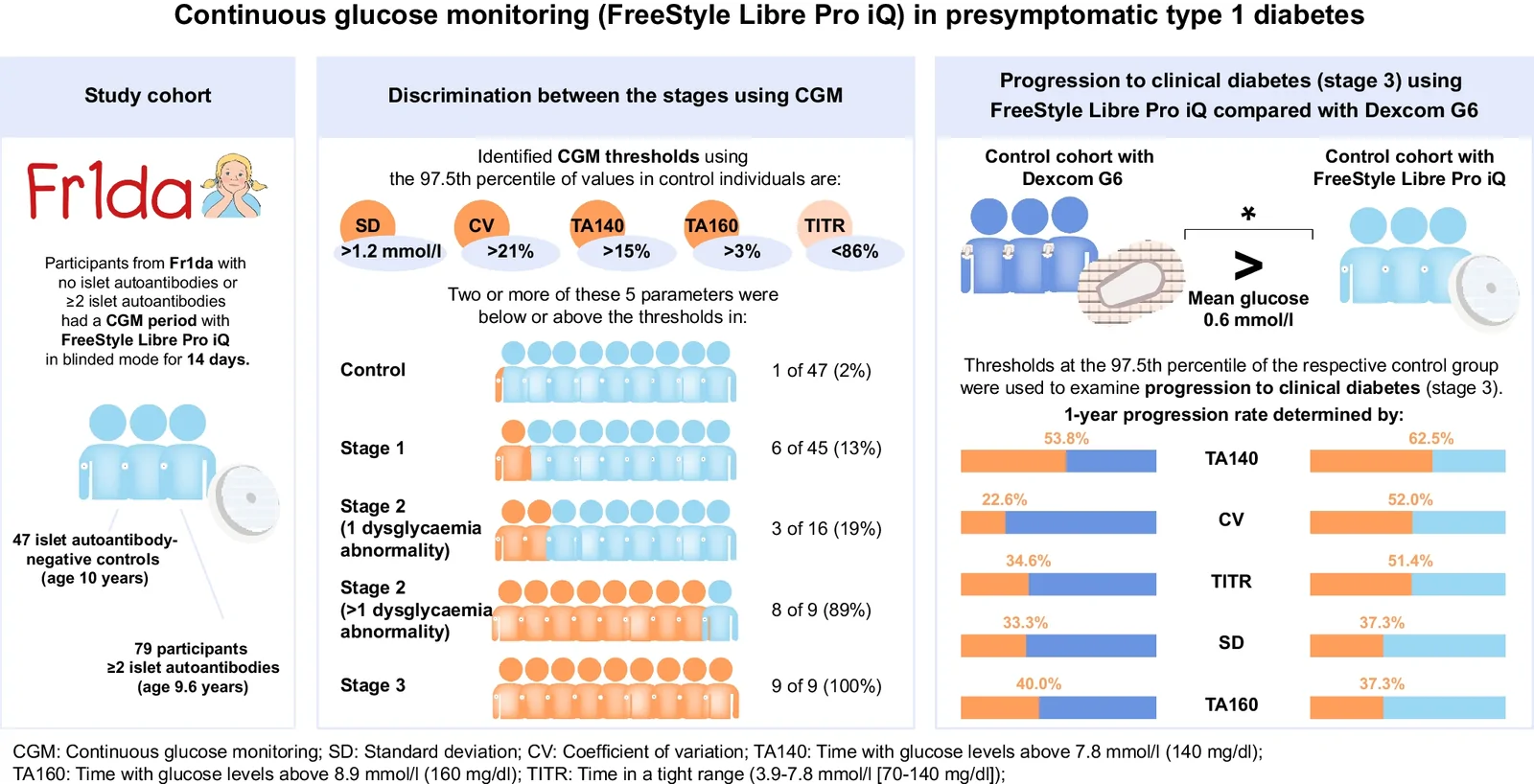

**Supplementary Information:**

The online version contains peer-reviewed but unedited supplementary material available at https://doi.org/10.1007/s00125-026-06852-w.

**Figure:**
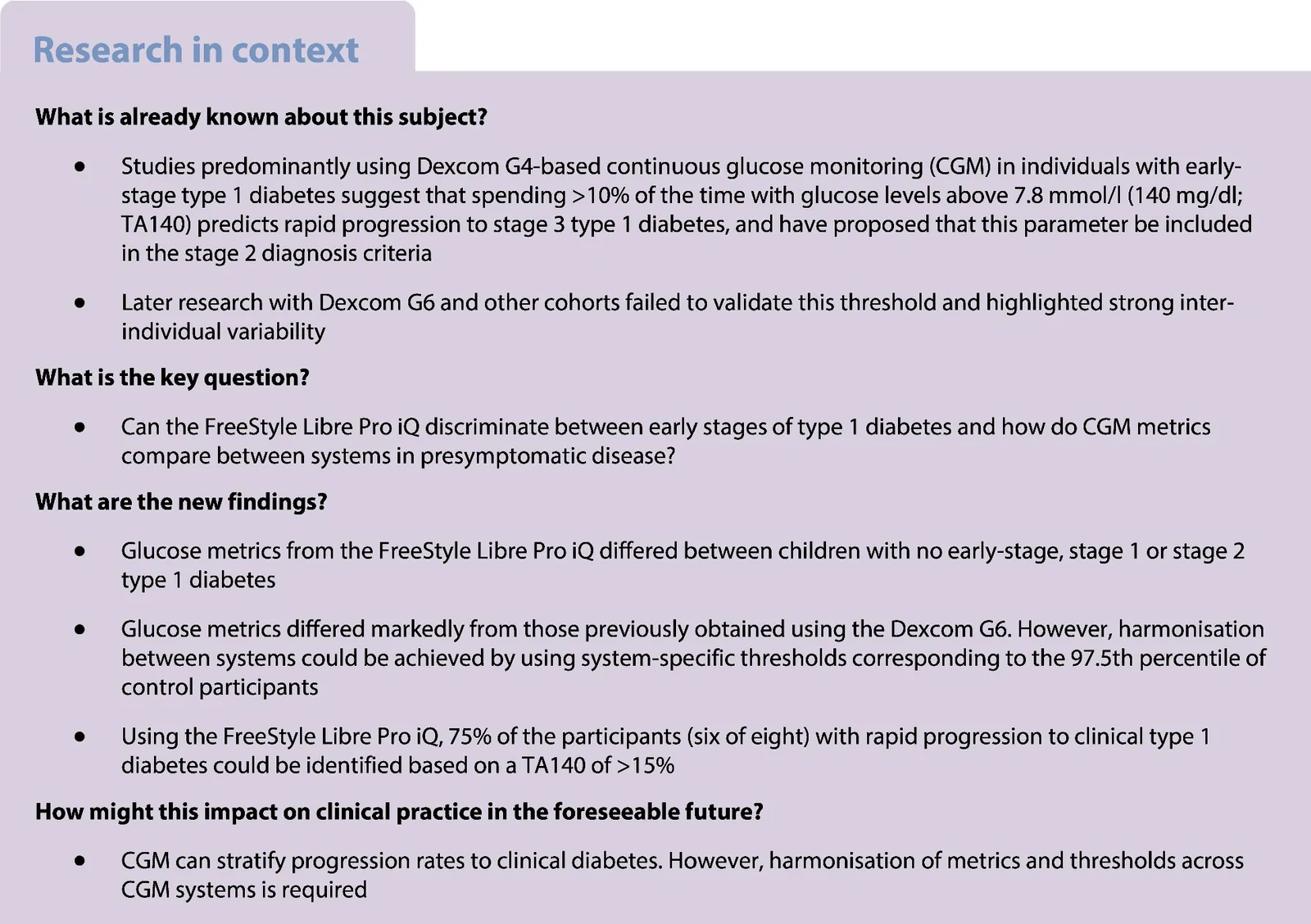

## Introduction

Type 1 diabetes is a chronic autoimmune disease characterised by immune-mediated destruction of the insulin-producing pancreatic beta cells [1]. The disease can now be diagnosed as early-stage type 1 diabetes years prior to the onset of clinical diabetes and the need for insulin replacement therapy [2, 3]. The classification of type 1 diabetes as either stage 1 or stage 2 [4, 5]—defined based on distinct presymptomatic phases without and with dysglycaemia, respectively—enables more precise stratification of the rate of disease progression and provides new perspectives to be considered when developing preventive and disease-modifying therapeutic strategies.

Diagnosing dysglycaemia requires the use of parameters that are currently obtained from an OGTT and HbA_1c_ testing. With the increasing identification of children, adolescents and adults in early-stage disease and the availability of the first immunomodulatory therapies that are capable of delaying clinical diabetes in stage 2, the following question arises: to what extent can continuous glucose monitoring (CGM) be meaningfully applied during this early phase? In particular, it remains to be clarified whether CGM-derived parameters can discriminate between different stages of presymptomatic type 1 diabetes and whether they are capable of predicting and monitoring disease progression, as well as guiding the timing and selection and assessing the efficacy of interventional therapies.

Over recent years, several studies have evaluated the ability of continuous glucose monitors, largely Dexcom devices, to stage and identify those who are likely to rapidly progress to clinical (stage 3) type 1 diabetes. The results have been heterogeneous. Early investigations employing the Dexcom G4 proposed a threshold of 10% of the time with glucose levels above >7.8 mmol/l (140 mg/dl; TA140) as a marker predictive of rapid progression to clinical diabetes [6, 7]. In contrast, our own studies using the Dexcom G6 [8], as well as investigations conducted in Australia [9], were unable to confirm these findings. Instead, it was observed that there is substantial inter-individual variability in glucose profiles and that meaningful predictive performance was achieved only through the use of other thresholds or composite scores that integrated multiple CGM-derived parameters.

Against this background, the present study is the first to evaluate an alternative, commonly used CGM system—namely the FreeStyle Libre Pro iQ, which is available as a blinded device—in presymptomatic type 1 diabetes. The aims of this study were to assess the ability of this device to discriminate between early stages of type 1 diabetes, to determine how well the device parameters may estimate the rate of disease progression and support the decision of whether to initiate disease-modifying therapies. It also aimed to explore the differences in performance between the FreeStyle Libre Pro iQ and the Dexcom G6.

## Methods

Children and adolescents participating in the FreeStyle Libre Pro iQ CGM study were enrolled from the Fr1da islet autoantibody screening programme [10]. Eligibility criteria for the screening programme included residence in Bavaria, Germany; age between 2 and 10 years at the time of screening; and written informed consent from at least one parent or legal guardian. The sex of participating children was determined based on parent-reported information; race or ethnicity data were not collected. Early-stage type 1 diabetes was diagnosed when individuals were positive for two or more islet autoantibodies against insulin, glutamic acid decarboxylase-65, insulinoma-associated antigen or zinc transporter 8 in screening and confirmatory samples, as previously described [10]. Families of children diagnosed with early-stage type 1 diabetes were invited to participate in a follow-up monitoring programme including an OGTT and HbA_1c_ testing at a clinical referral centre [10]. From December 2023 to December 2025, children and adolescents with early-stage type 1 diabetes aged ≥4 years who received care at the Munich clinical referral centre were asked to participate in this CGM study using the FreeStyle Libre Pro iQ CGM system. HbA_1c_ testing and an OGTT were also undertaken when the CGM device was placed. The median time from first screening to participation in the CGM study was 4.4 years (IQR 3.5–7.2). Additionally, children and adolescents participating in the Fr1da study who were negative for islet autoantibodies were enrolled as a control group. The CGM study was approved by the institutional review board at the Technical University of Munich. Written informed consent was obtained from participants or their parents or legal guardians. Additionally, participants in our previous CGM study using the Dexcom G6 [8] were followed up for the development of clinical (stage 3) type 1 diabetes and were used for comparison. Questionnaires on CGM wear experience were sent to participants with early-stage type 1 diabetes after completion of the first monitoring period with the FreeStyle Libre Pro iQ (electronic supplementary material [ESM] Methods).

### CGM

Blinded CGM systems were used to facilitate comparison with previous studies and to reduce the likelihood of behavioural changes in response to glucose excursions. Participants underwent a 14 day period of CGM. The sensor was mounted by trained staff and was placed on the back of the upper arm. Six different manufacturing lots of sensors were used throughout the project.

### Definition of control population and type 1 diabetes stages

Control participants were negative for islet autoantibodies at the time of CGM assessment. Stage 1 was defined by ADA criteria [5] as being positive for two or more islet autoantibodies and having normoglycaemia (ESM Methods). Stage 1 was also broken down into subgroups using the progression likelihood score (PLS), with stage 1a corresponding to a PLS of ≤4.0 and stage 1b corresponding to a PLS of >4.0 [11]. Stage 2 was defined as being positive for two or more islet autoantibodies and having dysglycaemia (ESM Methods). Stage 2 was further divided into subgroups as follows: those with one dysglycaemia abnormality (impaired fasting, intermediate or 2 h glucose during OGTT or impaired HbA_1c_) and those with more than one dysglycaemia abnormality [12]. OGTT and HbA_1c_ assessments were used solely when referring to dysglycaemia in the manuscript. Stage 3 (clinical type 1 diabetes) was defined by ADA criteria [5] (ESM Methods).

Serum C-peptide concentrations were measured by fluorescence enzyme immunoassays (AIA-360, Tosoh Bioscience South San Francisco, CA, USA). The Diabetes Prevention Trial-Type 1 Risk Score (DPTRS) and Index60, both of which are progression scores, were calculated as previously described [13, 14].

### Statistical analyses

The initial 12 h after each CGM dataset were excluded from the analysis, following previous recommendations [7, 15]. Implausible CGM readings (prolonged periods of values below 2.8 mmol/l [50 mg/dl]) were removed. Only datasets with more than 96 h remaining were included. For participants with repeated measurements, the first CGM measurement period was included in the overall analysis. The CGM parameters examined were the mean glucose concentration, SD, CV, TA140, time with glucose levels above 8.9 mmol/l (160 mg/dl; TA160), time with glucose levels below 3.9 mmol/l (70 mg/dl; TB70), time in tight range (3.9–7.8 mmol/l [70–140 mg/dl]; TITR) and mean glucose concentrations between 03:00 and 06:00. Statistical analyses were performed using R software, version 4.4.1 [16]. Variables were compared groups using the Kruskal–Wallis and Mann–Whitney *U* tests. Categorical data were compared using χ^2^ or Fisher's exact tests. Correlations were analysed using Pearson’s correlation coefficient. CGM parameters from this cohort were compared with CGM measurements from our previous CGM cohort that used Dexcom G6 [8] using *t* tests. Kaplan–Meier analyses were applied to estimate the rate of developing stage 3 type 1 diabetes with data censored according to the length of follow-up and the logrank test used to compare probabilities of outcomes in participants stratified for each variable (performed using R package survival, version 3.6.4 [17]). A two-tailed *p* value of <0.05 was considered significant.

## Results

In total, 139 children and adolescents were enrolled in the current CGM study to undergo 14 days of CGM using the blinded FreeStyle Libre Pro iQ. This included 50 control participants who were negative for islet autoantibodies and 89 participants with two or more islet autoantibodies (early-stage type 1 diabetes). Of these, two participants experienced sensor failures that prevented data collection, three did not return data and eight had fewer than 96 h of measurement, leaving 126 (91%) participants (73 female, median age 10.0 years [IQR 7.9–12.8]) for analysis (ESM Fig. 1). These included 47 participants without islet autoantibodies, 70 participants with early-stage type 1 diabetes and a further nine participants who were diagnosed with stage 3 onset at the time of the CGM study visit. Based on results from the HbA_1c_, OGTT and PLS assessments, 45 of the participants with early-stage type 1 diabetes had stage 1 (40 had stage 1a and five had stage 1b) and 25 had stage 2 (16 with one dysglycaemia abnormality and nine with more than one abnormality). A total of 26 participants underwent a second CGM period after a median follow-up of 9.2 months (IQR 5.4–12.4) and nine participants had more than two CGM periods.

Questionnaires regarding wearing the CGM device were returned by 78 participants (the median wearing time from CGM device placement was 90 days [IQR 20–149]). A preference for CGM was reported by 44 participants (60%) and a preference for OGTT was reported by 12 participants (16%) among the 73 who answered this question (*p*<0.0001), while 17 participants (23%) reported no preference (ESM Table 1). A similar distribution was observed among the 37 participants whose questionnaire responses were received within 90 days of CGM (ESM Table 1). Wearing the CGM device was considered either very comfortable or comfortable by 21 (27%) and 37 (47%) of the 78 participants, respectively, with no pain reported by 44 (56%) and hardly any pain reported by 26 (33%) participants. Activities during which the sensor was reported to be painful included sport and movement, sensor placement, sensor removal, initial or last days, dressing, sleeping or laying on the sensor, and accidentally knocking the sensor. Activities during which the sensor interfered with daily activities included dressing and undressing, sport, showering and sleeping (ESM Table 1).

### FreeStyle Libre Pro iQ CGM parameters in participants who were negative for islet autoantibodies and those with early-stage type 1 diabetes

**Fig. 1:**
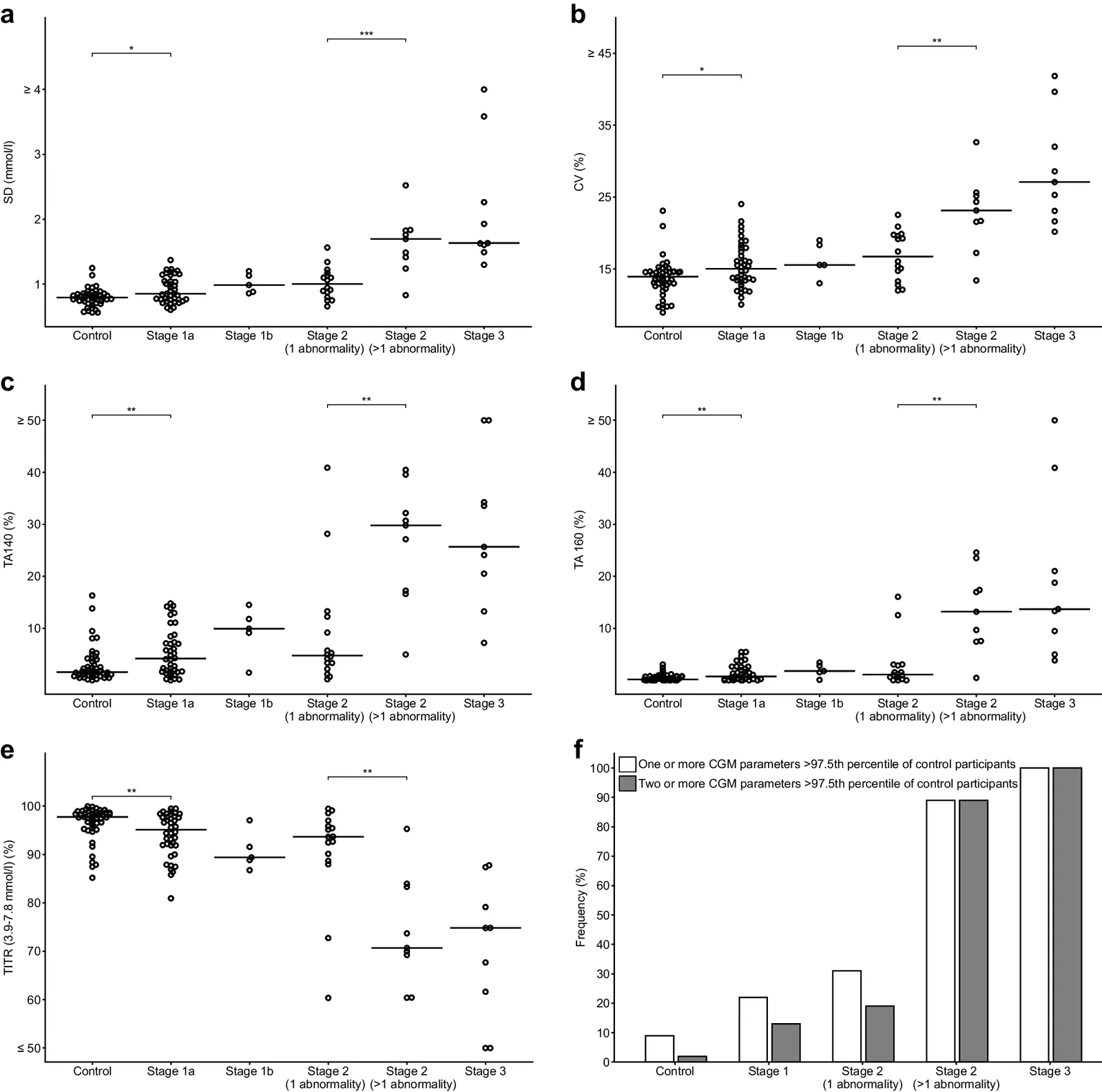
CGM metrics in the FreeStyle Libre Pro iQ cohort. Participants were stratified as control (negative for islet autoantibodies), stage 1a, stage 1b, stage 2 with one dysglycaemia abnormality, stage 2 with more than one dysglycaemia abnormality and newly diagnosed stage 3 type 1 diabetes at the time of CGM. The CGM parameters shown are (**a**) SD mmol/l, (**b**) CV (%), (**c**) TA140 (%), (**d**) TA160 (%) and (**e**) TITR (%). *p* values are given for the indicated comparisons. (**f**) The frequency of participants with at least one (open bars) and two or more (shaded bars) CGM parameters exceeding the 97.5th percentile of values in control participants, based on the parameters presented in (**a**)–(**e**). *p<0.05; **p<0.01; ***p<0.001

Multiple CGM parameters differed between participants without islet autoantibodies and participants with stage 1 or stage 2 type 1 diabetes (Table 1; Fig. 1). Compared with participants who were negative for islet autoantibodies, those with stage 1 type 1 diabetes differed in SD (*p*=0.0017), CV (*p*=0.0040), TA140 (*p*=0.0011), TA160 (*p*=0.0024) and TITR (*p*=0.0011). Differences were also observed between stage 1 and stage 2 type 1 diabetes for SD (*p*=0.0021), CV (*p*=0.0037), TA140 (*p*=0.013), TA160 (*p*=0.011) and TITR (*p*=0.014). These findings suggest progressive deterioration in glucose level profiles across early stages of type 1 diabetes. Additional heterogeneity was evident within stage 2 (Fig. 1, ESM Table 2). Participants with more than one dysglycaemia abnormality had higher values for most CGM metrics, including mean glucose (*p*=0.0006), SD (*p*=0.0008), CV (*p*=0.0035), TA140 (*p*=0.0027) and TA160 (*p*=0.0029), and had lower TITR (*p*=0.0028) than participants with only one dysglycaemia abnormality. Notably, participants with stage 2 type 1 diabetes and one dysglycaemia abnormality had CGM parameters similar to those of participants with stage 1 type 1 diabetes, whereas participants with more than one dysglycaemia abnormality had CGM values that did not differ from those observed in stage 3 type 1 diabetes. Sex differences were not observed.

**Table 1:** Study population by type 1 diabetes stage

Variable	Control	Stage 1	Stage 2	Stage 3	*p* value^a^	
	(*n*=47)	(*n*=45)	(*n*=25)	(*n*=9)	Control vs stage 1	Stage 1 vs stage 2
Age, years	10 (9–13)	9.1 (7.4–12.4)	10.3 (7.1–12.0)	9.3 (8.7–12.3)	0.25	0.84
Sex, male	18 (38.3)	18 (40.0)	12 (48.0)	5 (55.6)	1	0.69
No. of days of CGM data	15 (13.5–15)	14 (13–14)	14 (14–14)	13 (8–14)	0.0012	0.61
Mean glucose, mmol/l	5.8 (5.5–6.1)	6.0 (5.6–6.3)	6.3 (5.7–7.1)	7.1 (6.5–7.4)	0.062	0.071
SD, mmol/l	0.79 (0.73–0.85)	0.88 (0.77–1.13)	1.13 (0.90–1.49)	1.63 (1.61–2.26)	0.0017	0.0021
CV, %	13.9 (12.7–14.7)	15.4 (13.4–17.9)	19.2 (15.1–21.7)	27.1 (23.1–32.0)	0.0040	0.0037
TB70, %	0.1 (0.0–0.8)	0.2 (0.0–0.4)	0.4 (0.1–0.7)	1.4 (0.3–1.8)	0.88	0.15
TA140, %	1.6 (0.9–3.8)	4.8 (1.7–9.1)	9.2 (4.2–28.2)	25.7 (20.5–34.3)	0.0011	0.013
TA160, %	0.2 (0.0–0.7)	1.0 (0.1–2.6)	2.9 (0.6–12.5)	13.7 (9.5–21.0)	0.0024	0.011
Mean glucose, 03:00–06:00, mmol/l	5.2 (4.9–5.5)	5.3 (5.0–5.5)	5.4 (5.1–6.0)	6.1 (5.6–6.6)	0.33	0.15
>10% TA140	2 (4.3)	10 (22.2)	12 (48.0)	8 (88.9)	0.034	<0.0001
TITR, %	97.8 (95.7–98.6)	94.4 (91.9–97.8)	90.1 (72.7–95.3)	74.8 (61.6–79.1)	0.0011	0.014
HbA_1c_, %	Not available	5.4 (5.2–5.5)	5.7 (5.6–5.8)	6.4 (6.0–6.5)	Not available	<0.0001
HbA_1c_, mmol/mol		36 (33–37)	39 (38–40)	46 (42–48)		
C-peptide early, 30–0 min, nmol/l	Not available	0.93 (0.73–1.29)	0.70 (0.46–1.39)	0.30 (0.27–0.60)	Not available	0.23
Index60^b^	Not available	0.1 (−0.1–0.4)	1.3 (0.2–1.7)	2.8 (2.5–3.1)	Not available	0.0042
Index60^b^, positive		0 (0.0)	1 (5.3)	8 (88.9)		0.70
DPTRS^b^	Not available	5.8 (5.4–6.2)	7.2 (6.2–8.4)	10.3 (9.4–11.2)	Not available	0.0003
DPTRS^b^, positive		3 (7.5)	11 (57.9)	9 (100.0)		<0.0001

Data are reported as median (IQR) or *n* (%)

DPTRS [13]; Index60 [14]

^a^Mann–Whitney *U* test for continuous variables and χ^2^ test for categorical variables (uncorrected)

^b^Scores could not be calculated for five participants with stage 1 and six participants with stage 2 type 1 diabetes due to missing C-peptide measurements

### Discrimination between stages 1 and 2 using CGM

The CGM metrics that differed between stage 1 and stage 2 type 1 diabetes in the preceding analysis were carried forward. For these metrics, thresholds corresponding to the 97.5th percentile of the control participants were selected and evaluated for their ability to identify individuals with stage 2 type 1 diabetes. These thresholds were as follows: an SD of 1.2 mmol/l (21 mg/dl), a CV of 21%, a TA140 of 15%, a TA160 of 3% and a TITR of 86%. Employing these thresholds identified the following numbers of the 25 participants with stage 2 type 1 diabetes: 12 (48%) based on the SD, 8 (32%) based on the CV, 10 (40%) based on the TA140, 12 (48%) based on the TA160 and 10 (40%) based on the TITR (ESM Table 3). The specificity, defined as the proportion of those with stage 1 with a value under the 97.5th percentile of control participants, was 82.2% for the SD, 95.6% for the CV, 100% for the TA140, 84.4% for the TA160 and 95.6% for the TITR. The AUC in the receiver operating characteristic (ROC) analysis ranged from 0.68 for the TITR to 0.72 for the SD (ESM Fig. 2). Among participants with stage 2 type 1 diabetes, the five parameters also differed between those who had one dysglycaemia abnormality and those who had more than one dysglycaemia abnormality (ESM Table 4). The highest discrimination between stages 1 and 2 type 1 diabetes was obtained with the threshold of >15% for the TA140: none of the 45 participants with stage 1 type 1 diabetes exceeded this threshold, while 10 of the 25 participants with stage 2 type 1 diabetes exceeded it (*p*<0.0001). The thresholds of two or more of these five parameters were exceeded by 1 (2%) of the 47 control participants, 6 (13%) of the 45 participants with stage 1 type 1 diabetes (*p*=0.056) and 11 (44%) of the 25 participants with stage 2 type 1 diabetes (*p*=0.0077 vs stage 1), including three participants (19%) with one dysglycaemia abnormality and eight participants (89%) with more than one dysglycaemia abnormality. The thresholds of two or more of these five parameters were exceeded in all nine of the participants with stage 3 type 1 diabetes (*p*=0.0044 vs stage 2) (Fig. 1f). The thresholds of one or more parameters were exceeded by four (9%) of the control participants, 10 (22%) of those with stage 1 type 1 diabetes (*p*=0.085) and 13 (52%) of those with stage 2 type 1 diabetes (*p*=0.017 vs stage 1), including five participants (31%) with one dysglycaemia abnormality and eight participants (89%) with more than one dysglycaemia abnormality (Fig. 1f).

### Follow-up measurements

**Fig. 2:**
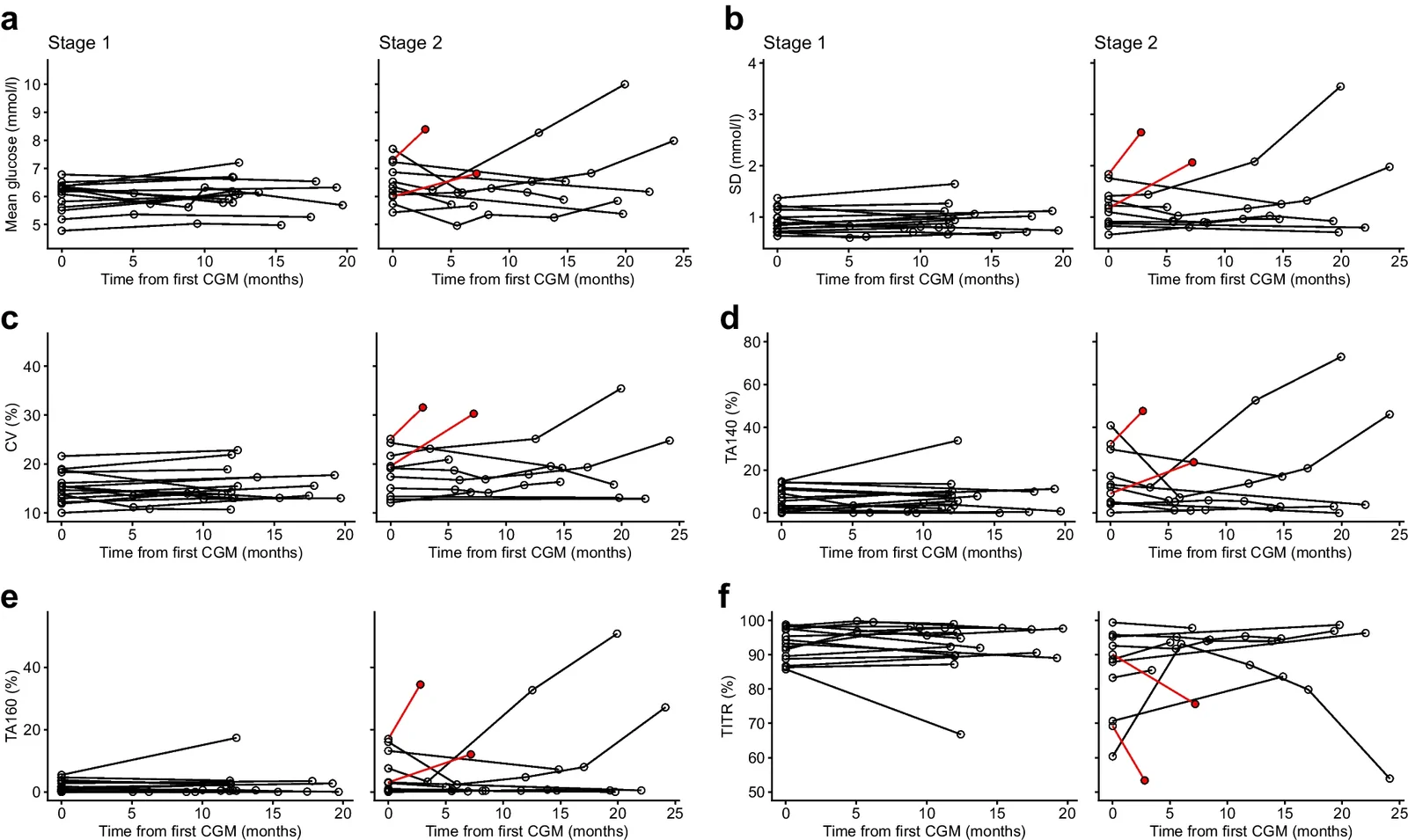
Sequential CGM metrics in participants with stage 1 (left, *n*=15) or stage 2 (right, *n*=11) type 1 diabetes who had at least two periods of CGM assessments: (**a**) mean glucose mmol/l, (**b**) SD mmol/l, (**c**) CV (%), (**d**) TA140 (%), (**e**) TA160 (%) and (**f**) TITR (%). Individual participants are connected by lines. Red lines indicate children who had progressed from stage 2 to stage 3 type 1 diabetes in the time between CGM measurements

Repeated CGM periods appeared relatively stable in participants with stage 1 type 1 diabetes, with the exception of one participant who had increases in their TA140 and TA160 values (Fig. 2). No change in stage was observed in this participant. In contrast, participants with stage 2 type 1 diabetes exhibited greater variability and ameliorations and deteriorations in the CGM metrics across repeated measurements. In particular, three participants showed substantial impairment in their CGM values. Two of these three participants progressed to stage 3. None of the remaining participants progressed to stage 3.

### Comparison between the FreeStyle Libre Pro iQ and the Dexcom G6

The Dexcom G6 device was previously used to report on 33 participants who were negative for islet autoantibodies and 64 participants with early-stage type 1 diabetes [8]. The study population was similar to the FreeStyle Libre Pro iQ cohort (ESM Table 5). An exploratory comparison of the CGM parameters between the two systems in participants without islet autoantibodies showed marked differences between the devices, with significantly higher CGM glycaemic metrics in the Dexcom G6 platform (ESM Fig. 3). The mean glucose measured using CGM in the participants without islet autoantibodies was, on average, 0.6 mmol/l (11.6 mg/dl) higher in the Dexcom G6 cohort than in the FreeStyle Libre Pro iQ cohort. For both cohorts, the mean glucose and the TA140 were correlated against parameters derived from the OGTT, including the AUC glucose, the early (30 min–baseline) C-peptide concentration during the OGTT, the DPTRS and Index60 in participants with early-stage type 1 diabetes (ESM Table 6). Correlation coefficients were higher for parameters obtained from the FreeStyle Libre Pro iQ than for those obtained from the Dexcom G6. Furthermore, the median difference between the estimated and actual HbA_1c_ was substantially less for the FreeStyle Libre Pro iQ (−0.2 mmol/mol [–0.01%]) than for the Dexcom G6 (+4.7 mmol/mol [0.43%]; *p*<0.0001, ESM Fig. 4). An overestimation of the HbA_1c_ by 4.7 mmol/mol (0.43%) corresponded to a mean glucose of 0.7 mmol/l (12.3 mg/dl). Adjusting all glucose values obtained from the Dexcom G6 by −0.6 mmol/l (11.6 mg/dl) led to convergence for most parameters (ESM Fig. 5).

### Progression to clinical diabetes (stage 3)

**Fig. 3:**
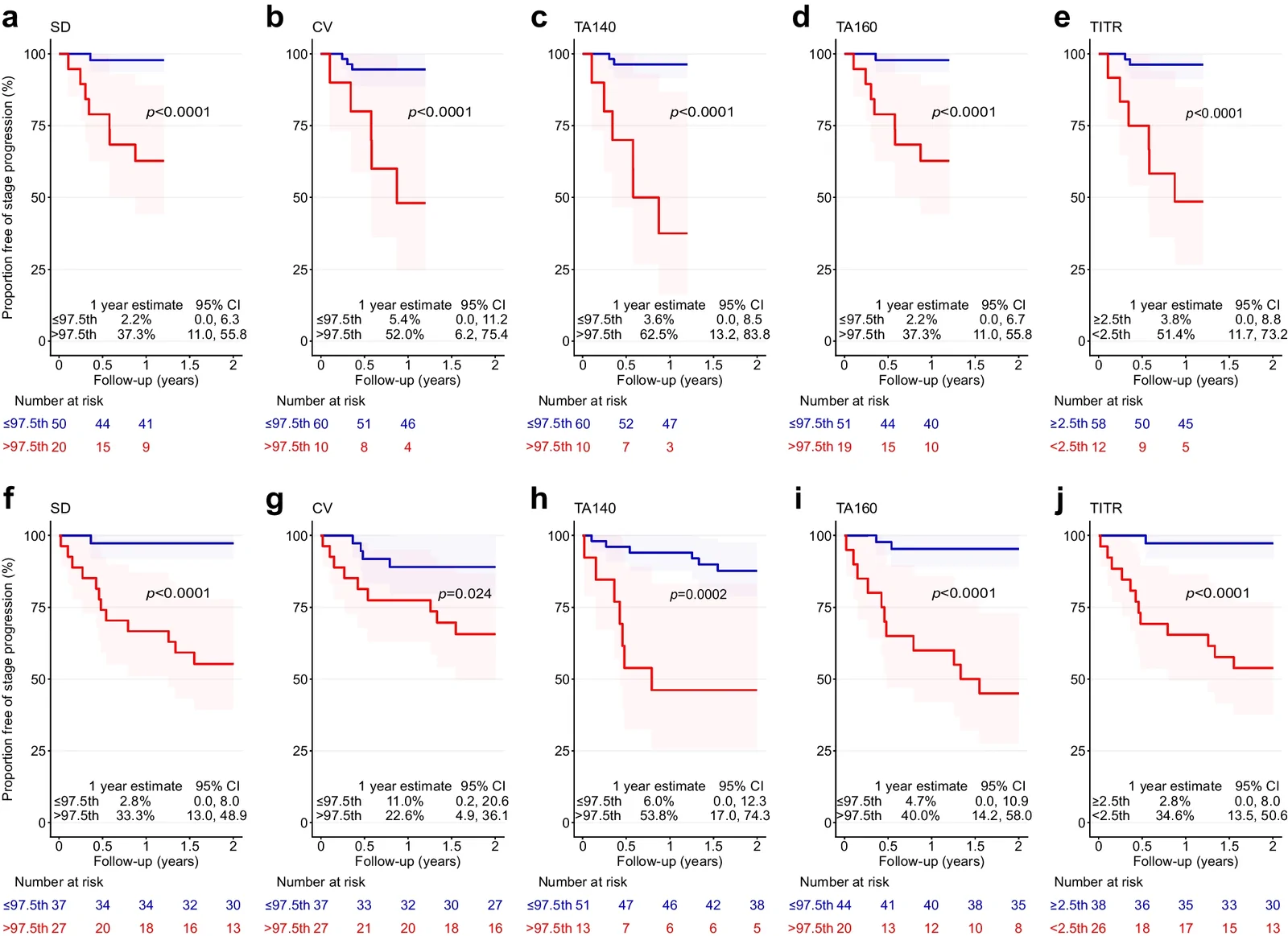
CGM parameter stratification of the progression rate to stage 3 type 1 diabetes. Kaplan–Meier analysis of the proportion remaining without stage 3 type 1 diabetes after categorisation of participants in the FreeStyle Libre Pro iQ cohort (**a**–**e**) and in the Dexcom G6 cohort (**f**–**j**) as above (red lines) or below (blue lines) the 97.5th percentile of control cohorts for SD (**a**, **f**), CV (**b**, **g**), TA140 (**c**, **h**) and TA160 (**d**, **i**) and above and below the 2.5th percentile for TITR (**e**, **j**). The thresholds correspond to 1.2 mmol/l (21 mg/dl) for SD, 21% for CV, 15% for TA140, 3% for TA160 and 86% for TITR in the FreeStyle Libre Pro iQ cohort and 1.2 mmol/l (21 mg/dl) for SD, 17% for CV, 36% for TA140, 12% for TA160 and 60% for TITR in the Dexcom G6 cohort. Shaded areas represent the 95% CIs

Progression to clinical diabetes occurred in eight participants with early-stage type 1 diabetes who had measurements with the FreeStyle Libre Pro iQ during a median observation time of 1.6 years (total observation time: 94.5 years). Among those with early-stage type 1 diabetes who had Dexcom G6 measurements, 16 of 64 progressed to clinical diabetes during a median observation time of 2.7 years (total observation time: 144.4 years). The annualised progression rate in the two studies was similar (8.5% vs 11.1%; *p*=0.55). Within each cohort, progression was stratified by stages 1 and 2 as defined by HbA_1c_ and OGTT assessments (ESM Fig. 6). The ROC curve analysis showed differences between the participants who progressed to stage 3 within 1 year and those who did not progress for each of the CGM parameters analysed, with the AUC ranging from 0.9 to 0.92 for the FreeStyle Libre Pro iQ and from 0.72 to 0.9 for the Dexcom G6 (ESM Fig. 7). We used thresholds at the 97.5th percentile of the control groups for the SD, CV, TA140, TA160 and TITR to examine progression rates to clinical diabetes (stage 3). For the Dexcom G6 cohort, these corresponded to 1.2 mmol/l (21 mg/dl), 17%, 36%, 12% and 60%, respectively. Each of the parameters significantly stratified progression using either the FreeStyle Libre Pro iQ or the Dexcom G6 platform (Fig. 3). One-year progression ranged from 37.3% (95% CI 11.0, 55.8) for the SD and TA160 to 62.5% (95% CI 13.2, 83.8) for the TA140 in the FreeStyle Libre Pro iQ cohort and from 22.6% (95% CI 4.9, 36.1) for the CV to 53.8% (95% CI 17.0, 74.3) for the TA140 in the Dexcom G6 cohort. The sensitivity for identifying those who progressed to clinical diabetes within 1 year ranged from 75% to 100% for the FreeStyle Libre Pro iQ and from 50% to 100% for the Dexcom G6 (ESM Table 7). In an exploratory analysis, we also used the adjusted glucose values of the Dexcom G6 CGM measurements, with thresholds derived from the 97.5th percentile of the combined 80 control participants without islet autoantibodies from the FreeStyle Libre Pro iQ cohort and Dexcom G6 cohort (ESM Fig. 8). These corresponded to 1.1 mmol/l (20 mg/dl) for the SD, 23% for the CV, 20% for the TA140, 5% for the TA160 and 75% for the TITR. Again, this stratified progression in both cohorts with similar risks and sensitivity.

Finally, the previously proposed threshold of 10% for the TA140 was evaluated. In total, 22 individuals with early-stage type 1 diabetes (10 with stage 1 and 12 with stage 2) from the FreeStyle Libre Pro iQ cohort had a TA140 of >10%. The 1 year progression among these 22 individuals as identified by the 10% TA140 threshold was 27.6% (95% CI 6.1, 44.1; sensitivity 75%) compared with 62.5% (95% CI 13.2, 83.8; sensitivity 75%) when the 97.5th percentile (a TA140 of >15%) was applied. Using the combined cohorts and adjusted glucose values, 49 of the 134 participants had a TA140 of >10%. The 1 year progression in these 49 participants was 32.8% (95% CI 18.2, 44.7; sensitivity 89%).

## Discussion

This study evaluated the use of the FreeStyle Libre Pro iQ in blinded mode to characterise glycaemic parameters in children with early-stage type 1 diabetes. Several CGM-derived metrics differed between children without islet autoantibodies, children with stage 1 type 1 diabetes and children with stage 2 type 1 diabetes, and these identified the majority of participants who progressed to clinical (stage 3) type 1 diabetes within 1 year. These findings support the concept that CGM systems capture progressive disturbances in glycaemic regulation across presymptomatic stages of type 1 diabetes. The questionnaire results showed that CGM was better accepted by participants than an OGTT, supporting a future shift towards more intensive CGM use for staging in individuals’ interests.

Study strengths include the careful metabolic staging and substaging of participants, including the performance of the OGTT and CGM at the same visit. The study allowed the comparison of two different CGM platforms in similar cohorts, which is an issue of major relevance for clinical implementation. Finally, it included control groups, which enabled the derivation of platform-specific percentiles that also allowed the validation of findings. There were also several limitations. Subgroup size remained relatively small, particularly for stages 1 and 2 type 1 diabetes. The prospective follow-up was relatively short for definitive progression analyses. In addition, the Dexcom G6 and FreeStyle Libre Pro iQ datasets were derived from different participant cohorts, precluding direct within-person sensor comparison. Therefore, although the observed differences between devices were substantial and clinically meaningful, some of these differences may also reflect cohort-related variation. In addition, methodological differences, including sensor insertion sites and sensor wear duration, may have contributed to the observed differences in results. The utility of percentile thresholds across CGM systems should, therefore, be confirmed in larger prospective studies, ideally including the simultaneous or sequential use of multiple CGM systems in the same participants. Preferences for OGTT vs CGM were assessed using a questionnaire, but this evaluation was added later in the study, resulting in some retrospective assessments. Furthermore, data reflected a collection of responses from children and parents. The study was performed in children and adolescents, and the findings may not reflect those in adults. Finally, factors affecting glucose levels such as age, obesity, insulin sensitivity and genetic determinants of glucose metabolism were not assessed.

Although multiple CGM parameters differed between control participants, participants with stage 1 type 1 diabetes and participants with stage 2 type 1 diabetes, substantial inter-individual variation was present within each group. CGM values potentially consistent with dysglycaemia were observed across all groups, including participants without islet autoantibodies. The most consistently elevated values were seen in participants with stage 2 type 1 diabetes and multiple dysglycaemia abnormalities, whose CGM measures were indistinguishable from those of individuals with new-onset stage 3 type 1 diabetes. In contrast, participants with stage 2 type 1 diabetes with a single dysglycaemia abnormality had CGM values similar to those observed in stage 1 type 1 diabetes. Overall, incorporating CGM into staging, or replacing OGTT and/or HbA_1c_ assessment with CGM, would likely identify additional or different individuals as having ‘stage 2 disease’, with potential implications for therapy eligibility. Simply adding more parameters to the definition of stage 2 type 1 diabetes has the danger of reducing stage 2 progression rates. Furthermore, some of the analysed CGM metrics are strongly correlated with each other, limiting comparisons of parameter performance. Such considerations are important and will require more data from multiple systems and longer follow-up before CGM can be incorporated into staging definitions.

As we had previously conducted a similar study using the Dexcom G6 platform, we were able to compare values and performances between the two systems. Marked differences in measurements were observed between the systems. Compared with the Dexcom G6, the FreeStyle Libre Pro iQ showed closer HbA_1c_ estimates to venous values, stronger correlations with OGTT-derived metabolic measures and the previously developed progression scores (DPTRS [13] and Index60 [14]), more stable repeated measurements in participants with stage 1 type 1 diabetes and fewer technical failures (9% vs 18% by the Dexcom G6). Variations between systems have been previously reported in adults with type 1 diabetes [18]. Taken together, these data strongly suggest that one should be cautious in applying absolute CGM thresholds across different sensor systems. This has direct implications for the use of thresholds such as a TA140 of >10% as a surrogate definition for stage 2 type 1 diabetes or as a marker to identify individuals who are at high risk of progression who may be candidates for disease-modifying therapies. Our results indicate that such thresholds are likely to be platform dependent and, therefore, may result in inconsistent classification across studies and clinical settings. For the FreeStyle Libre Pro iQ, a TA140 threshold of 15% was both specific for stage 2 type 1 diabetes and associated with rapid progression to stage 3 type 1 diabetes, and appeared more appropriate than the 10% threshold previously suggested for the Dexcom G4. Nevertheless, in view of the differences observed between systems and the frequent evolution of CGM models along with new generations and systems, it is unlikely that the threshold observed with the FreeStyle Libre Pro iQ can be applied universally. System variation represents a major hindrance to the use of CGM in early-stage type 1 diabetes classification and monitoring.

To address the challenge posed by systematic differences between systems, we introduced a platform-adapted percentile-based approach using values in individuals without islet autoantibodies to define CGM parameter abnormalities. Notably, these percentile-derived cut-offs differed substantially between the Dexcom G6 and the FreeStyle Libre Pro iQ systems, confirming that direct numerical equivalence between sensor systems cannot be assumed. Despite these differences in absolute thresholds, percentile-based classification identified comparable progression rates to clinical diabetes across systems using multiple parameters. In participants with values above the 97.5th percentile of control participants, 1 year progression was comparable to or higher than the rates observed when participants were classified as having stage 2 type 1 diabetes using HbA_1c_ and OGTT values. We chose the 97.5th percentile of controls as a threshold for CGM parameters to favour specificity, but we recognise that this may not be the optimal percentile for staging or risk stratification and that other percentile-based thresholds should be evaluated. As an alternative, we also used an adjusted glucose value for the Dexcom G6 cohort, which partially improved comparability. This exploratory approach offered a possibility to use universal thresholds to identify a subgroup of participants who rapidly progressed to clinical diabetes. Other algorithms to harmonise values derived from different systems may also be considered [19, 20].

In summary, this study extends previous work on CGM in presymptomatic type 1 diabetes in several important ways. To our knowledge, it is the first study to evaluate the FreeStyle Libre Pro iQ in this context, the first to compare CGM-derived glucose levels across different sensor platforms and the first to demonstrate that the number of HbA_1c_- and OGTT-based dysglycaemia abnormalities within stage 2 type 1 diabetes can be distinguished using CGM. Despite differences in multiple CGM parameters between stages and substages, the study shows marked variation in the classification of early-stage (stages 1 and 2) type 1 diabetes by CGM compared with established measures, and marked variation in values obtained from different CGM systems. Overall, these findings support the use of CGM as a promising tool for refined staging and risk stratification in early type 1 diabetes, while underscoring the need for system- and generation-specific interpretation before CGM metrics can be used reliably in clinical trials or for therapeutic decision-making.

## Supplementary Information

Below is the link to the electronic supplementary material.

ESM (PDF 481 KB)

## Data Availability

All datasets generated and analysed during the current study are not publicly available but are available from the corresponding author on reasonable request. Requests should include a project description outlining the proposed use of the data and the scientific rationale. Data access is subject to completion of a data use agreement with Helmholtz Munich.

## Abbreviations

CGM: Continuous glucose monitoring
DPTRS: Diabetes Prevention Trial-Type 1 Risk Score
PLS: Progression likelihood score
ROC: Receiver operating characteristic
TA140: Time with glucose levels above 7.8 mmol/l (140 mg/dl)
TA160: Time with glucose levels above 8.9 mmol/l (160 mg/dl)
TB70: Time with glucose levels below 3.9 mmol/l (70 mg/dl)
TITR: Time in tight range (3.9–7.8 mmol/l [70–140 mg/dl])
