# Supplementary material for: Continuous glucose monitoring using the FreeStyle Libre Pro iQ in presymptomatic type 1 diabetes
Source: Diabetologia. 2026 Sep 7;69(12):3512–22. doi: 10.1007/s00125-026-06852-w (PMC13714109; doi:10.1007/s00125-026-06852-w)
Supplement: Supplementary file 1 [file 125_2026_6852_MOESM1_ESM.pdf]

SUPPLEMENTARY MATERIAL

Continuous glucose monitoring using the FreeStyle Libre Pro iQ in presymptomatic type 1 diabetes

Andreas Weiss[1,2], Elisabeth Huber[1], Franziska Reinmüller[1], Annette Munzinger[1], Ezio Bonifacio[3,4], Anette-Gabriele Ziegler[1,2,5]

[1] Institute of Diabetes Research, Helmholtz Munich, German Research Center for Environmental Health, Neuherberg, Germany

[2] German Center for Diabetes Research (DZD e.V.), Neuherberg, Germany

[3] Center for Regenerative Therapies Dresden, Technische Universität Dresden, Dresden, Germany

[4] Paul Langerhans Institute Dresden of the Helmholtz Munich at University Hospital Carl Gustav Carus, Faculty of Medicine, Technische Universität Dresden, Dresden, Germany

[5] Technical University of Munich, School of Medicine and Health, Forschergruppe Diabetes, TUM University Hospital, Munich, Germany

Andreas Weiss and Elisabeth Huber are joint first authors.

Table of Contents

**ESM Methods**	Definitions of normoglycaemia, dysglycaemia and clinical diabetes.	4
**ESM Methods**	CGM questionnaires	4
**ESM Table 1.**	Responses from a questionnaire survey in 78 participants of the FreeStyle Libre Pro iQ study cohort.	5
**ESM Table 2.**	CGM parameters in control participants and in participants with early stage type 1 diabetes.	6
**ESM Table 3.**	Sensitivity and 1-specificity of CGM parameters for discriminating stage 2 from stage 1.	7
**ESM Table 4.**	Sensitivity and 1-specificity of CGM parameters for discriminating stage 2 with more than one dysglycemia abnormality from one dysglycemia abnormality.	8
**ESM Table 5.**	Comparison of the Dexcom G6 and the FreeStyle Libre Pro iQ study cohorts.	9
**ESM Table 6.**	Correlations with OGTT metrics, early C-peptide, and progression scores for the Dexcom G6 and the FreeStyle Libre Pro iQ.	10
**ESM Table 7.**	Sensitivity, specificity, PPV and NPV of CGM parameters for discriminating children who progressed to stage 3 T1D within one year against children who had at least one year follow-up without progression to stage 3 T1D in that period.	11
**ESM Fig. 1.**	Consort diagram of study participants enrolled in the FreeStyle Libre Pro iQ CGM study.	12
**ESM Fig. 2.**	Receiver Operator Characteristic (ROC) curves for distinguishing stage 2 from stage 1 type 1 diabetes.	13

**ESM Fig. 3.**	Comparison of the CGM metrics of the control participants measured by the Dexcom and FreeStyle Libre Pro iQ.	14
**ESM Fig. 4.**	Comparison between venous blood HbA1c and estimated HbA1c by Dexcom G6 and by FreeStyle Libre Pro iQ.	15
**ESM Fig. 5.**	Comparison of the CGM metrics of the control participants measured by the Dexcom G6 and FreeStyle Libre Pro iQ after correction of Dexcom G6 values for glucose difference.	16
**ESM Fig. 6.**	Kaplan Meier curves for progression to stage 3 T1D from stage 1 and stage 2	17
**ESM Fig. 7.**	Receiver Operator Characteristic (ROC) curves for one year progression.	18
**ESM Fig. 8.**	CGM parameter stratification of progression rate to stage 3 type 1 diabetes.	19

ESM Methods

CGM questionnaires

From August 2024, questionnaires on CGM wear experience were sent to participants with early-stage type 1 diabetes after completion of the first monitoring period with the Freestyle Libre Pro iQ. This included participants who had their first monitoring prior to August 2024. Responses to questions 2–4 were collected using a 5-point Likert scale, with an optional free-text field for additional comments. Families were encouraged to have participants complete the questionnaire whenever possible; however, it was not recorded whether responses were provided by the participant or a parent. Questionnaire responses were returned by mail.

Definitions of normoglycaemia, dysglycaemia and clinical diabetes.

The ADA criteria used to define normoglycaemia were fasting plasma glucose <100 mg/dL (5.6 mmol/L), 2-hour plasma glucose <140 mg/dL (7.8 mmol/L), plasma glucose <200 mg/dL (11.1 mmol/L) at 30, 60, or 90 minutes, and HbA1c <39 mmol/mol (5.7%).

The ADA criteria used to define dysglycaemia were fasting plasma glucose of 100-125 mg/dL (5.6-6.9 mmol/L) and/or impaired 2-hour plasma glucose of 140-199 mg/dL (7.8-11.0 mmol/L), and/or plasma glucose ≥200 mg/dL (11.1 mmol/L) at 30, 60, or 90 minutes, and/or HbA1c 39-47 mmol/mol (5.7-6.4%).

The ADA criteria used to define stage 3 (clinical) diabetes were fasting plasma glucose ≥126 mg/dL (7.0 mmol/L) or a 2-hour plasma glucose of ≥200 mg/dL (11.1 mmol/L) in an OGTT; or HbA1c >48 mmol/mol (6.5%); or in children with classic symptoms of hyperglycaemia, a random plasma glucose of >200 mg/dL (11.1 mmol/L) in the absence of unequivocal hyperglycaemia. In the absence of unequivocal hyperglycaemia, diagnosis required two abnormal results from different tests, which may be obtained at the same time, or the same test at two different time points.

ESM Table 1. Responses from a questionnaire survey in 78 participants of the FreeStyle Libre Pro iQ study cohort.

**Question**	Total	Responses received < 90 days after OGTT and CGM
What would you rather do, if you could choose?	n = 73	n = 37
CGM	44 (60%)	23 (62%)
OGTT	12 (16%)	6 (16%)
Either CGM of OGTT (no preference)	17 (23%)	8 (22%)
How comfortable was wearing the CGM?	n = 78	n = 41
Very Comfortable	21 (27%)	14 (34%)
Comfortable	37 (47%)	18 (44%)
Moderately comfortable	18 (23%)	7 (17%)
Uncomfortable	1 (1%)	1 (2%)
Very Uncomfortable	1 (1%)	1 (2%)
How painful was wearing the CGM?	n = 78	n = 41
No pain	44 (56%)	26 (63%)
Hardly any pain	26 (33%)	11 (27%)
A little pain	7 (9%)	3 (7%)
Moderate pain	1 (1%)	1 (2%)
Strong pain	0	0
Which activities or situations: sport and movement (4), sensor placement (4), sensor removal (4), initial or last days (4), dressing (3), sleeping or laying on sensor (3), knocking the sensor (2), loose sensor (2), itch (1), shower (1)[a]		
Did the sensor disturb you in your everyday activities?	n = 78	n = 41
Never	35 (45%)	18 (44%)
Seldom	30 (38%)	18 (44%)
Sometimes	13 (17%)	4 (10%)
Often	1 (1%)	1 (2%)
Always	0	0
Which activities: dressing and undressing (14), sport (12), shower (4), sleeping (1)[a]		

[a]Number in parentheses refer to number of responses indicating a specific activity or situation

ESM Table 2. CGM parameters in control participants and in participants with early stage type 1 diabetes.

CGM Metrics	Control	Stage 1 n = 45		Stage 2 n = 25		Stage 3[a] n = 9
		1a n = 40	1b n = 5	1 abnormality n = 16	>1 abnormality n = 9	
Age, years, median (IQR)	10 (9-13)	9.2 (7.5-12.4)	8.2 (5.5-13.1)	9.5 (6.9-13.3)	11.4 (9.4-11.9)	9.3 (8.7-12.3)
Sex, male, n (%)	18 (38.3)	14 (35.0)	4 (80.0)	8 (50.0)	4 (44.4)	5 (55.6)
No. of days of CGM data, median (IQR)	15 (13.5-15)	14 (12-14)	15 (14-15)	14 (13-14)	14 (14-14)	13 (8-14)
Mean glucose (mmol/l), median (IQR)	5.8 (5.5-6.1)	6.0 (5.6-6.3)	6.3 (6.2-6.3)	5.9 (5.5-6.3)	7.2 (6.5-7.3)	7.1 (6.5-7.4)
SD (mmol/l), median (IQR)	0.79 (0.73-0.85)	0.86 (0.76-1.07)	0.99 (0.88-1.13)	1.01 (0.86-1.14)	1.69 (1.42-1.83)	1.63 (1.61-2.26)
CV (%), median (IQR)	13.9 (12.7-14.7)	15.0 (13.4-17.5)	15.6 (15.6-18.3)	16.7 (14.4-19.6)	23.1 (21.6-25.2)	27.1 (23.1-32.0)
TB70 (%), median (IQR)	0.1 (0.0-0.8)	0.2 (0.0-0.3)	0.2 (0.1-1.8)	0.5 (0.1-1.7)	0.1 (0.1-0.5)	1.4 (0.3-1.8)
TA140 (%), median (IQR)	1.6 (0.9-3.8)	4.2 (1.7-7.6)	9.9 (9.1-11.8)	4.8 (3.0-9.9)	29.8 (17.2-32.2)	25.7 (20.5-34.3)
TA160 (%), median (IQR)	0.2 (0.0-0.7)	0.7 (0.1-2.3)	1.8 (1.6-2.8)	1.1 (0.5-2.9)	13.2 (7.6-17.4)	13.7 (9.5-21.0)
Mean glucose (0300-0600 h) (mmol/l), median (IQR)	5.2 (4.9-5.5)	5.2 (5.0-5.6)	5.4 (5.3-5.5)	5.1 (5.0-5.5)	6.2 (5.6-7.0)	6.1 (5.6-6.6)
>10% TA140, n (%)	2 (4.3)	8 (20.0)	2 (40.0)	4 (25.0)	8 (88.9)	8 (88.9)
TITR (%), median (IQR)	97.8 (95.7-98.6)	95.1 (91.9-97.8)	89.4 (88.8-91.6)	93.7 (89.8-96.1)	70.7 (69.2-83.3)	74.8 (61.6-98.6)
HbA1c (%), median (IQR)	not available	5.4 (5.2-5.5)	5.6 (5.5-5.6)	5.7 (5.6-5.8)	5.7 (5.6-6.0)	6.4 (6.0-6.5)
HbA1c [mmol/mol], median (IQR)		36 (33-37)	38 (37-38)	39 (38-40)	39 (38-42)	46 (42-48)
C-Peptide early (30 min. – 0 min.) (nmol/l), median (IQR)	not available	0.85 (0.66-1.17)	0.45 (0.36-0.97)	0.72 (0.57-1.51)	0.42 (0.27-0.94)	0.30 (0.27-0.60)
Index60, median (IQR)	not available	0.1 (-0.1-0.4)	0.7 (-0.7-1.2)	0.3 (-0.3-1.3)	1.7 (1.4-2.0)	2.8 (2.5-3.1)
Index60, positive, n (%)		0 (0.0)	0 (0.0)	0 (0.0)	1 (14.3)	8 (88.9)
DPTRS, median (IQR)	not available	5.7 (5.4-6.1)	7.2 (6.2-7.3)	6.3 (5.7-7.1)	8.5 (8.4-8.5)	10.3 (9.4-11.2)
DPTRS, positive, n (%)		1 (2.7)	2 (66.7)	4 (33.3)	7 (100.0)	9 (100.0)

[a]newly diagnosed stage 3; CGM, continuous glucose monitoring; IQR, interquartile range; SD, standard deviation; CV, coefficient of variation; TB, time below; TA, time above; TITR, time in tight range (3.9 – 7.8 mmol/l); DPTRS, Diabetes Prevention Trial-Type 1 Risk Score [13]; Index60 [14];

ESM Table 3. Sensitivity and 1-specificity of CGM parameters for discriminating stage 2 from stage 1.

CGM parameter[a]	Stage 2 (n= 25) N pos (Sensitivity, %)	Stage 1 (n = 45) N pos (1-specificity, %)	*P* value
SD (> 1.2 mmol/l)	12 (48%)	8 (17.8)	0.016
CV (>21%)	8 (32%)	2 (4.4)	0.0051
TA140 (>15%)	10 (40%)	0 (0)	<0.0001
TA160 (>3%)	12 (48%)	7 (15.6)	0.0082
TITR (<86%)	10 (40%)	2 (4.4)	0.0006
One or more CGM parameters[b]	13 (52%)	10 (22.2)	0.024
Two or more CGM parameters[b]	11 (44%)	6 (13.3)	0.010
Three or more CGM parameters[b]	10 (40%)	2 (4.4)	0.0006

[a]Thresholds defined as the 97.5th percentile of controls without islet autoantibodies (FreeStyle Libre Pro iQ only) for SD, CV, TA140, TA160, and the 2.5th centile for TITR.

[b]Refers to one or more, two or more, three or more of the five CGM parameters included in the table

ESM Table 4. Sensitivity and 1-specificity of CGM parameters for discriminating stage 2 with more than one dysglycemia abnormality from one dysglycemia abnormality.

CGM parameter[a]	>1 dysglycemia (n= 9) N pos (Sensitivity, %)	1 dysglycemia (n = 16) N pos (1-specificity, %)	*P* value
SD (>1.2 mmol/L)	8 (88.9)	4 (25.0)	0.0080
CV (>21%)	7 (77.8)	1 (6.3)	0.0012
TA140 (>15%)	8 (88.9)	2 (12.5)	0.0009
TA160 (>3%)	8 (88.9)	4 (25.0)	0.0080
TITR (<86%)	8 (88.9)	2 (12.5)	0.0009
One or more CGM parameters[b]	8 (88.9)	5 (31.3)	0.019
Two or more CGM parameters[b]	8 (88.9)	3 (18.8)	0.0030
Three or more CGM parameters[b]	8 (88.9)	2 (12.5)	0.0009

[a]Thresholds defined as the 97.5th percentile of controls without islet autoantibodies (FreeStyle Libre Pro iQ only) for SD, CV, TA140, TA160, and the 2.5th centile for TITR.

[b]Refers to one or more, two or more, three or more of the five CGM parameters included in the table

ESM Table 5. Comparison of the Dexcom G6 and the FreeStyle Libre Pro iQ study cohorts.

	Dexcom G6<br>n=97	FreeStyle Libre Pro iQ<br>n=126
Age, years, median (IQR)	10 (7-12)	10 (8-13)
Sex, male, n (%)	40 (41.2)	53 (42.1)
Time from first Fr1da screening, years, median (IQR)	4.7 (2.8-6.6)	4.4 (3.5-7.2)
No. of days of CGM data, median (IQR)	9.3 (8.6-9.4)	14 (13-15)

ESM Table 6. Correlations with OGTT metrics, early C-peptide, and progression scores for the Dexcom G6 and the FreeStyle Libre Pro iQ.

	Dexcom G6		FreeStyle Libre Pro iQ without stage 3	
	Correlation[e]	*P* value	Correlation[e]	*P* value
CGM Mean glucose vs AUC OGTT[a]	0.312	0.013	0.320	0.0068
CGM Mean glucose vs early OGTT C-Peptide (30 min – 0 min values)	-0.142	0.32	-0.233	0.074
CGM Mean glucose vs DPTRS[b]	0.300	0.036	0.425	0.0008
CGM Mean glucose vs Index60[c]	0.207	0.15	0.366	0.0044
CGM TA140[d] vs DPTRS[b]	0.313	0.029	0.592	<0.0001
CGM TA140[d] vs Index60[c]	0.203	0.15	0.489	<0.0001

[a] Area under the curve for glucose in the OGTT
[b] Diabetes Prevention Trial-Type 1 Risk Score defined by glucose and c-peptide values in the OGTT, age and BMI (reference 13 in main manuscript)
[c] Index60 is defined by fasting and 60-minute c-peptide vaues and 60-minute glucose values in the OGTT (reference 14 in main manuscript)
[d] Time above 7.8 mmol/l
[e] Correlations were performed using Pearson's correlation coefficient

ESM Table 7. Sensitivity, specificity, PPV and NPV of CGM parameters for discriminating children who progressed to stage 3 T1D within one year against children who had at least one year follow-up without progression to stage 3 T1D in that period.

CGM parameter[a]	Sensitivity (%)	Specificity (%)	PPV (%)	NPV (%)
**Freestyle Libre Pro iQ**				
SD (>1.2 mmol/L)	87.5	82.0	35.0	97.6
CV (>21%)	62.5	92.0	50.0	93.5
TA140 (>15%)	75.0	94.0	60.0	95.7
TA160 (>3%)	87.5	80.0	36.8	97.5
TITR (<86%)	75.0	90.0	50.0	95.6
Any of the above CGM parameters	87.5	76.0	36.8	97.4
Two or more CGM parameters	87.5	84.0	46.7	97.7
Three or more CGM parameters	75.0	90.0	54.5	95.7
**Dexcom G6**				
SD (>1.2 mmol/L)	90.0	65.4	33.3	97.1
CV (>17%)	60.0	61.5	22.6	87.5
TA140 (>36%)	70.0	88.5	53.8	93.5
TA160 (>12%)	80.0	76.9	40.0	95.0
TITR (<60%)	90.0	67.3	34.6	97.1
One or more CGM parameters[b]	100.0	57.7	31.3	100.0
Two or more CGM parameters[b]	90.0	65.4	33.3	97.1
Three or more CGM parameters[b]	80.0	75.0	38.1	95.1

[a]Thresholds defined as the 97.5th percentile of controls without islet autoantibodies (FreeStyle Libre Pro iQ only) for SD, CV, TA140, TA160, and the 2.5th centile for TITR.

[b]Refers to one or more, two or more, three or more of the five CGM parameters included in the table

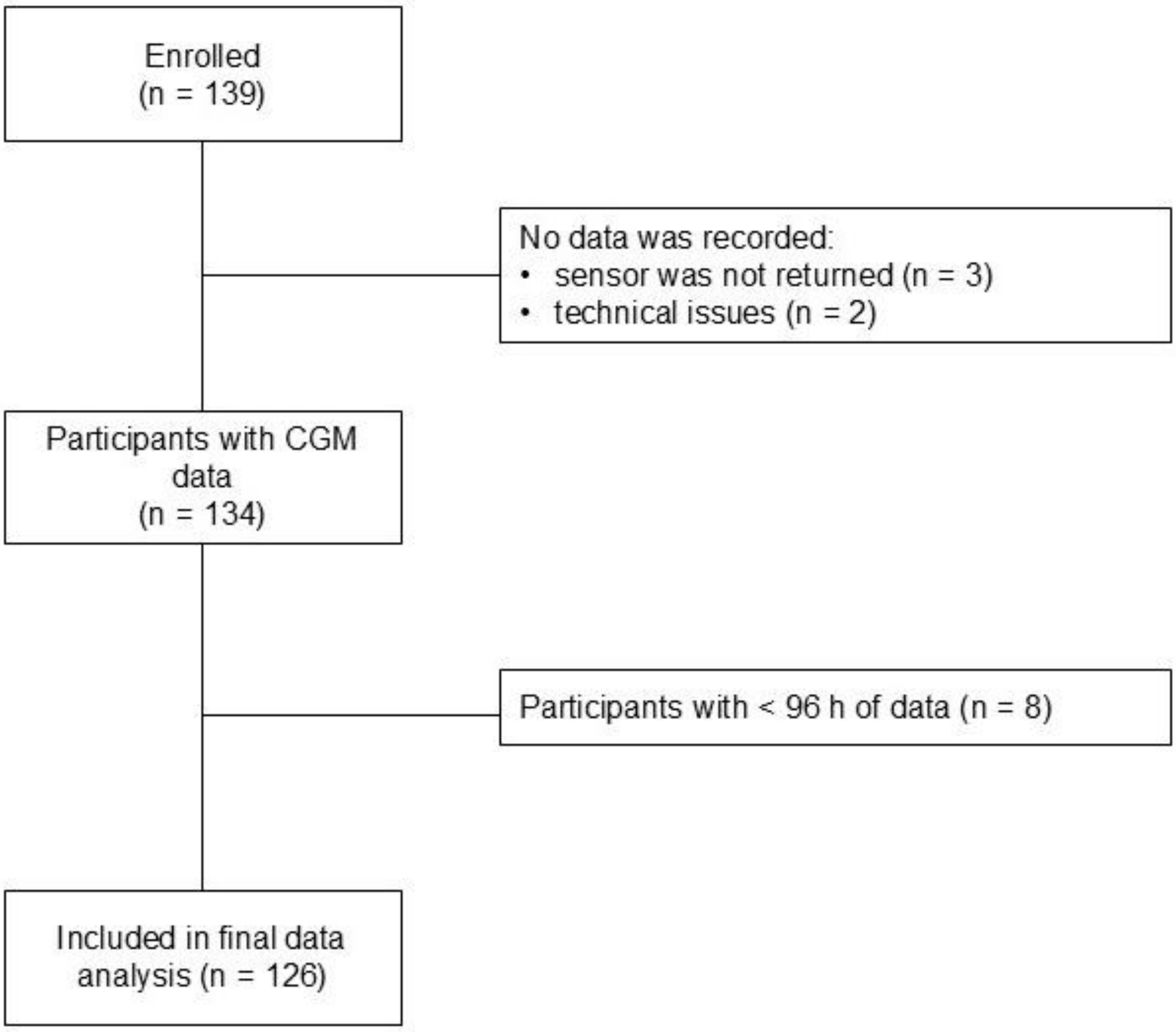

ESM Fig. 1. Consort diagram of study participants enrolled in the FreeStyle Libre Pro iQ CGM study.

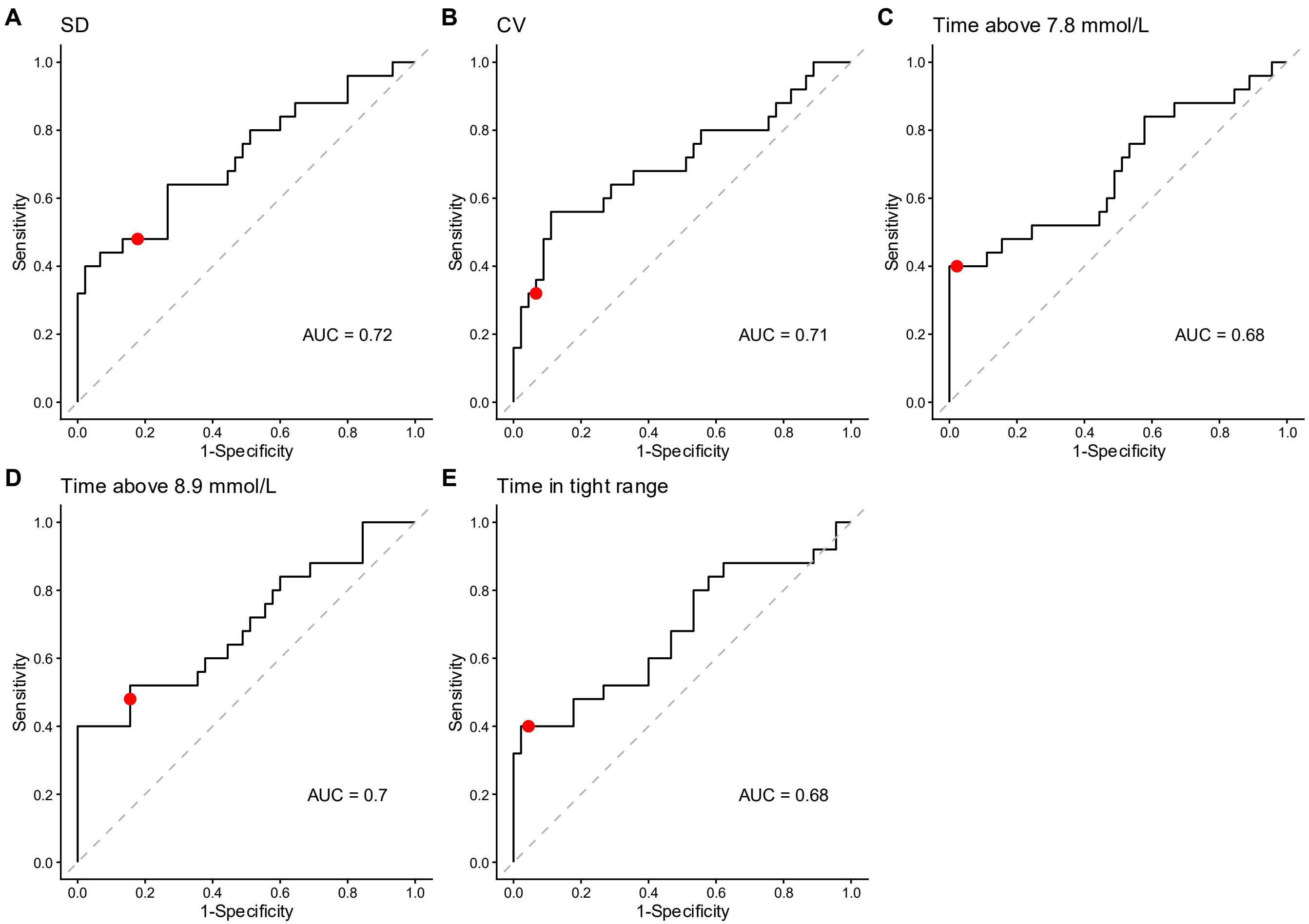

ESM Fig. 2. Receiver Operator Characteristic (ROC) curves for distinguishing stage 2 from stage 1 type 1 diabetes. Curves are shown for the indicated CGM parameters in the FreeStyle Libre Pro iQ cohort (A-E). Participants were categorized into those with stage 2 defined by HbA1c and OGTT (sensitivity, n = 25) and those with stage 1 (1 – specificity, n = 45). The red filled circle represents the values corresponding to the CGM parameter 97.5th centile of controls.

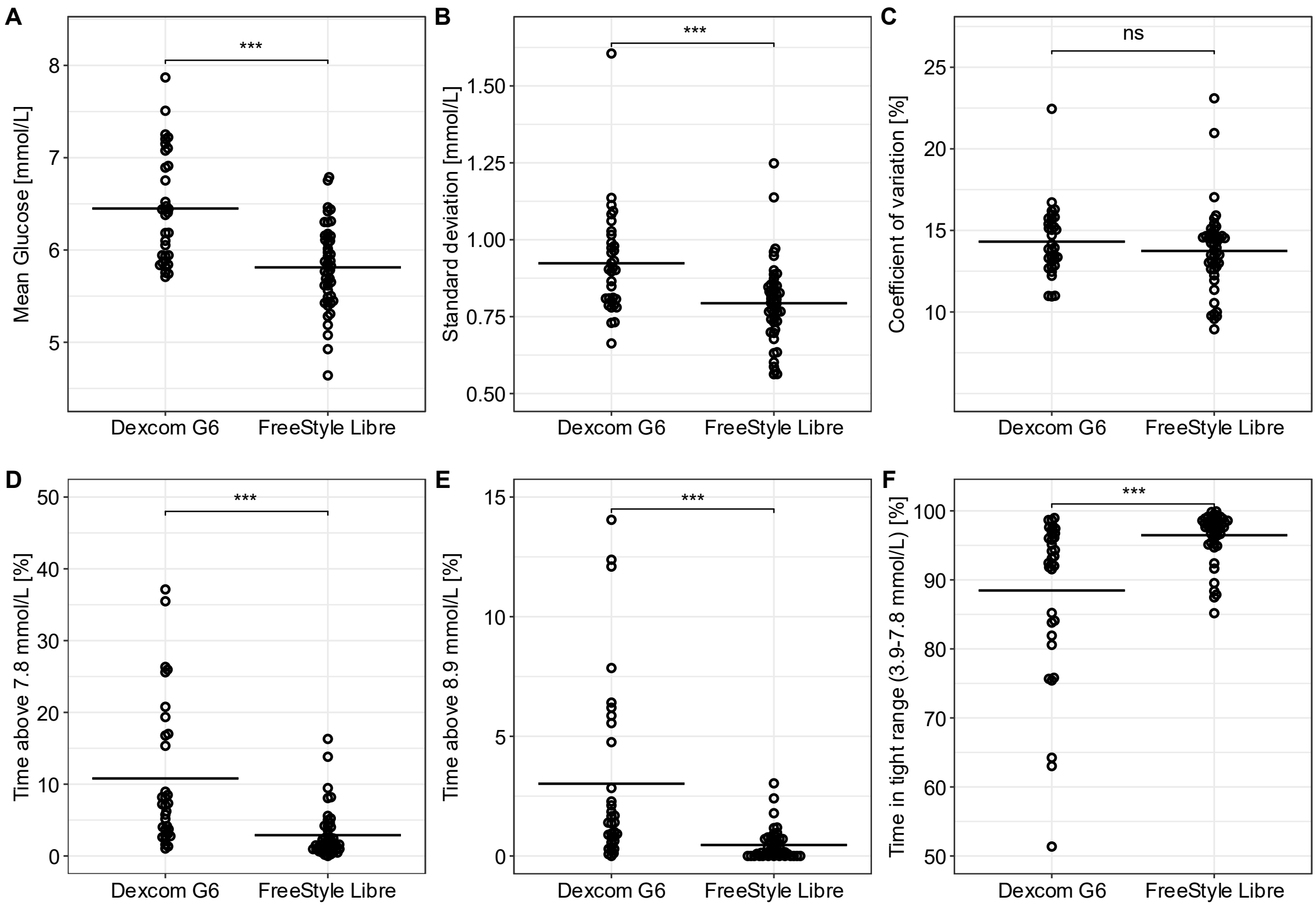

ESM Fig. 3. Comparison of the CGM metrics of the control participants measured by the Dexcom G6 (left, n=33 [8]) and FreeStyle Libre Pro iQ (right, n=47). CGM parameters shown are (A) mean glucose (mmol/l), (B) standard deviation (mmol/l), (C) coefficient of variation (%), (D) TA140 (time above 7.8 mmol/l) (%), (E) TA160 (time above 8.9 mmol/l) (%), and (F) time in tight range (%). ns, non-significant; **, p<0.01; ***, p<0.001

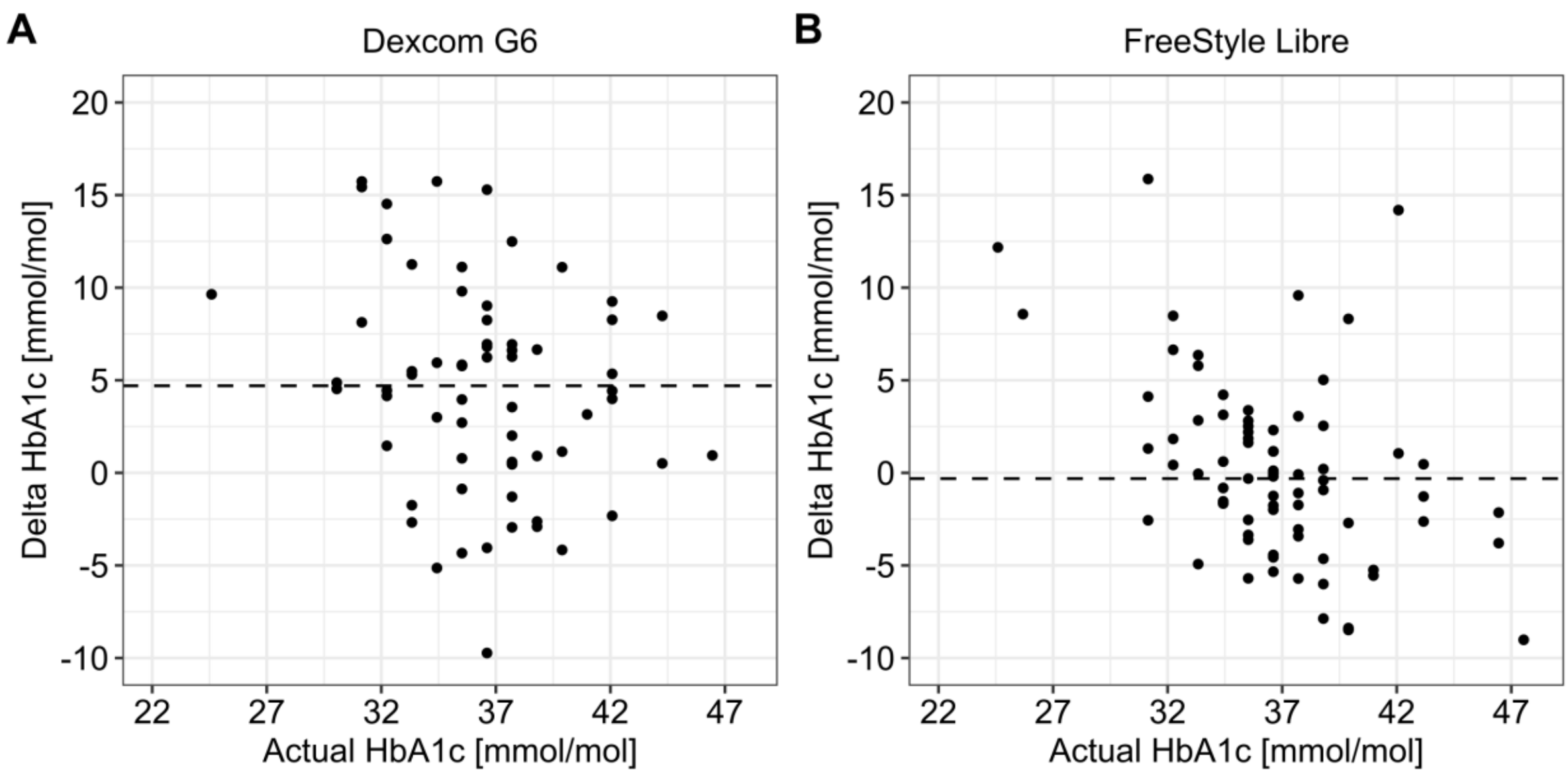

ESM Fig. 4. Comparison between venous blood HbA1c (x axis) and estimated HbA1c [1] by (A) Dexcom G6 and (B) by FreeStyle Libre Pro iQ shown as a delta to the venous blood measurement (y axis).

1. Nathan DM, Kuenen J, Borg R, Zheng H, Schoenfeld D, Heine RJ (2008) A1c-Derived Average Glucose Study Group. Translating the A1C assay into estimated average glucose values. Diabetes Care 31(8):1473-8. https://doi.org/10.2337/dc08-0545

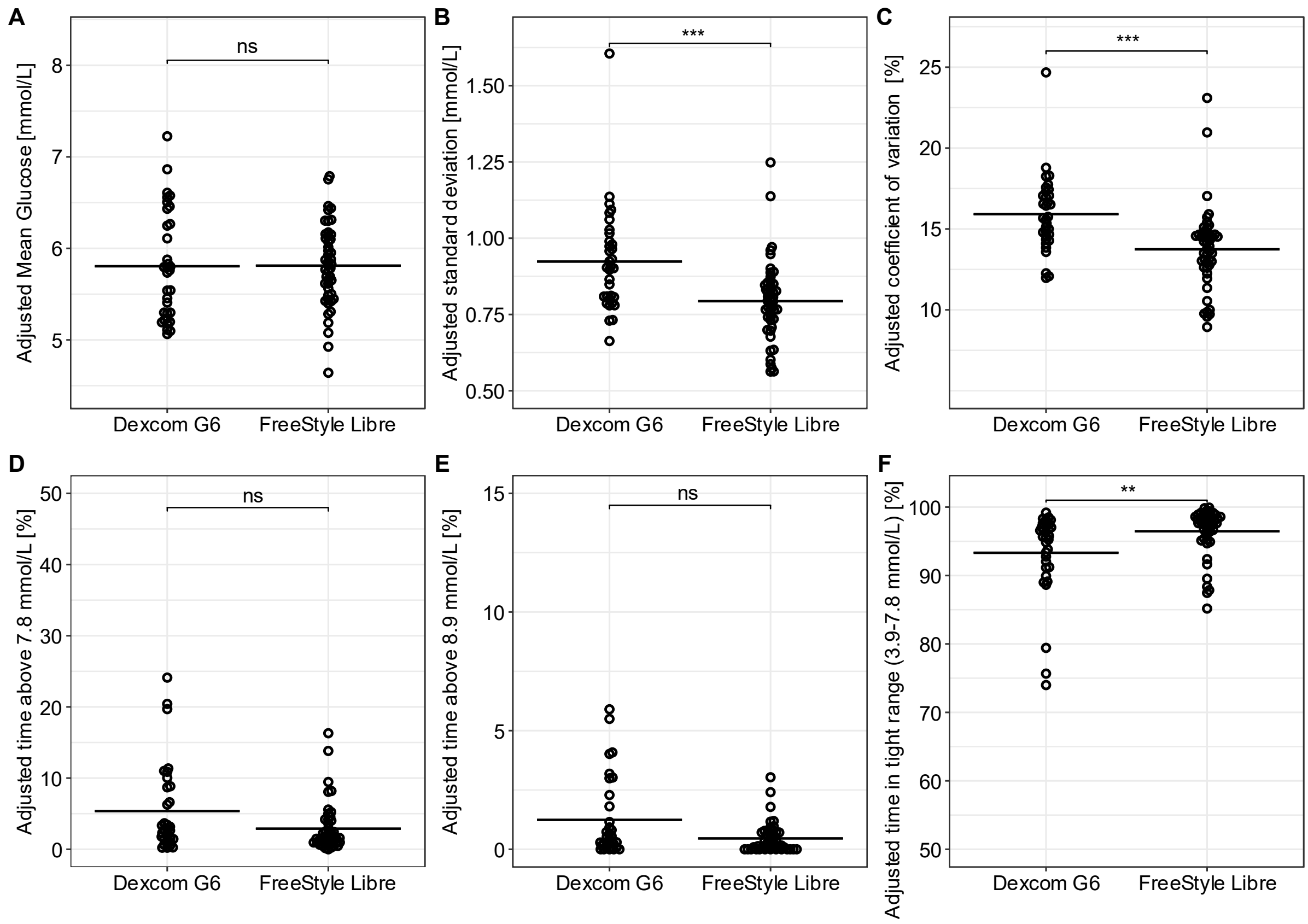

ESM Fig. 5. Comparison of the CGM metrics of the control participants measured by the Dexcom G6 (left, n=33 [8]) and FreeStyle Libre Pro iQ (right, n=47) after correction of Dexcom G6 values for glucose difference. CGM parameters shown are (A) mean glucose (mmol/L), (B) standard deviation (mmol/L), (C) coefficient of variation (%), (D) time above 7.8 mmol/L (%), (E) time above 8.9 mmol/L (%), and (F) time in tight range (%). ns, non-significant; **, p<0.01; ***, p<0.001

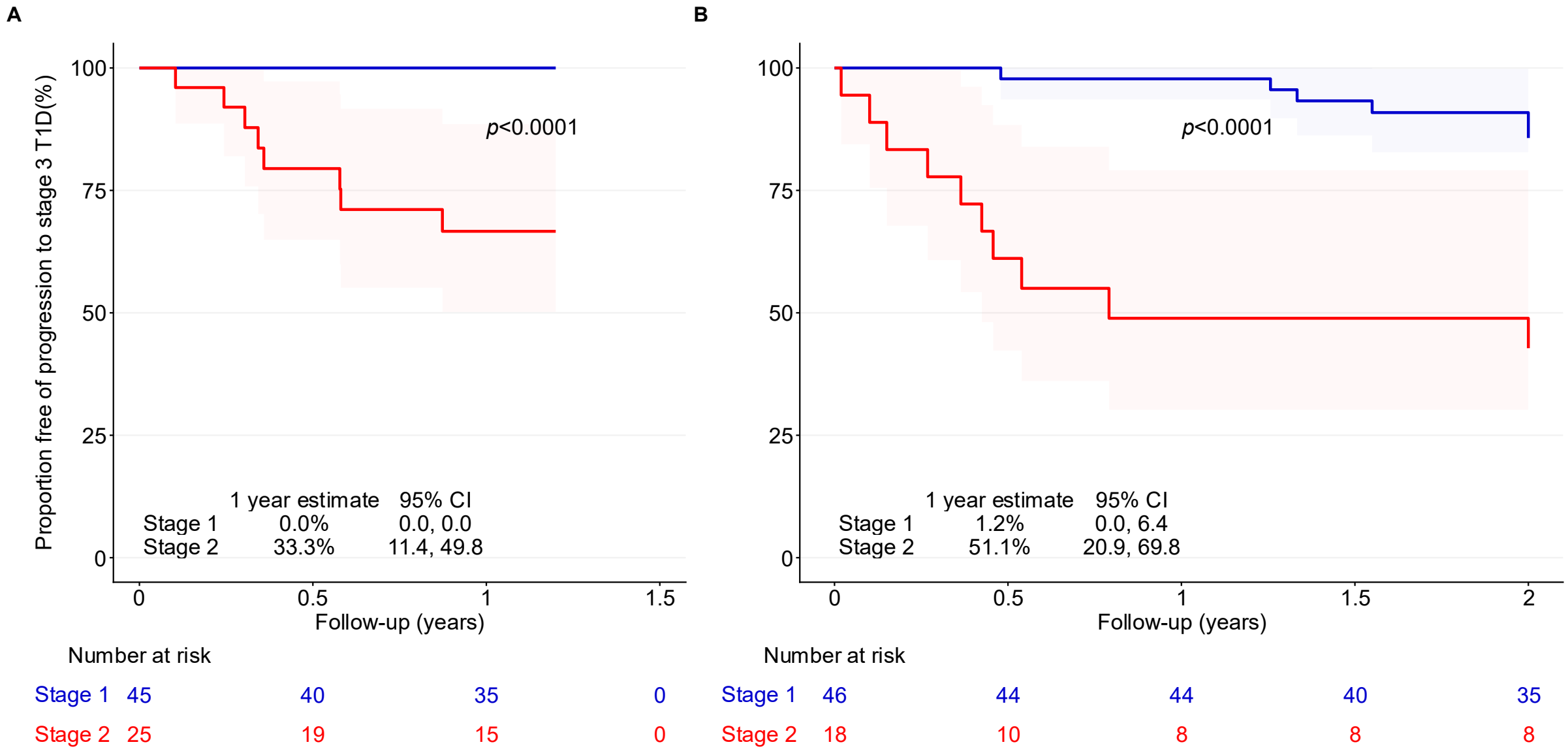

ESM Fig. 6 Kaplan Meier curves of progression to Stage 3 Type 1 Diabetes in Stage 1 (blue) and Stage 2 (red) in the Freestyle Libre Pro iQ cohort (A) and Dexcom G6 cohort (B). Shaded areas mark 95% confidence intervals.

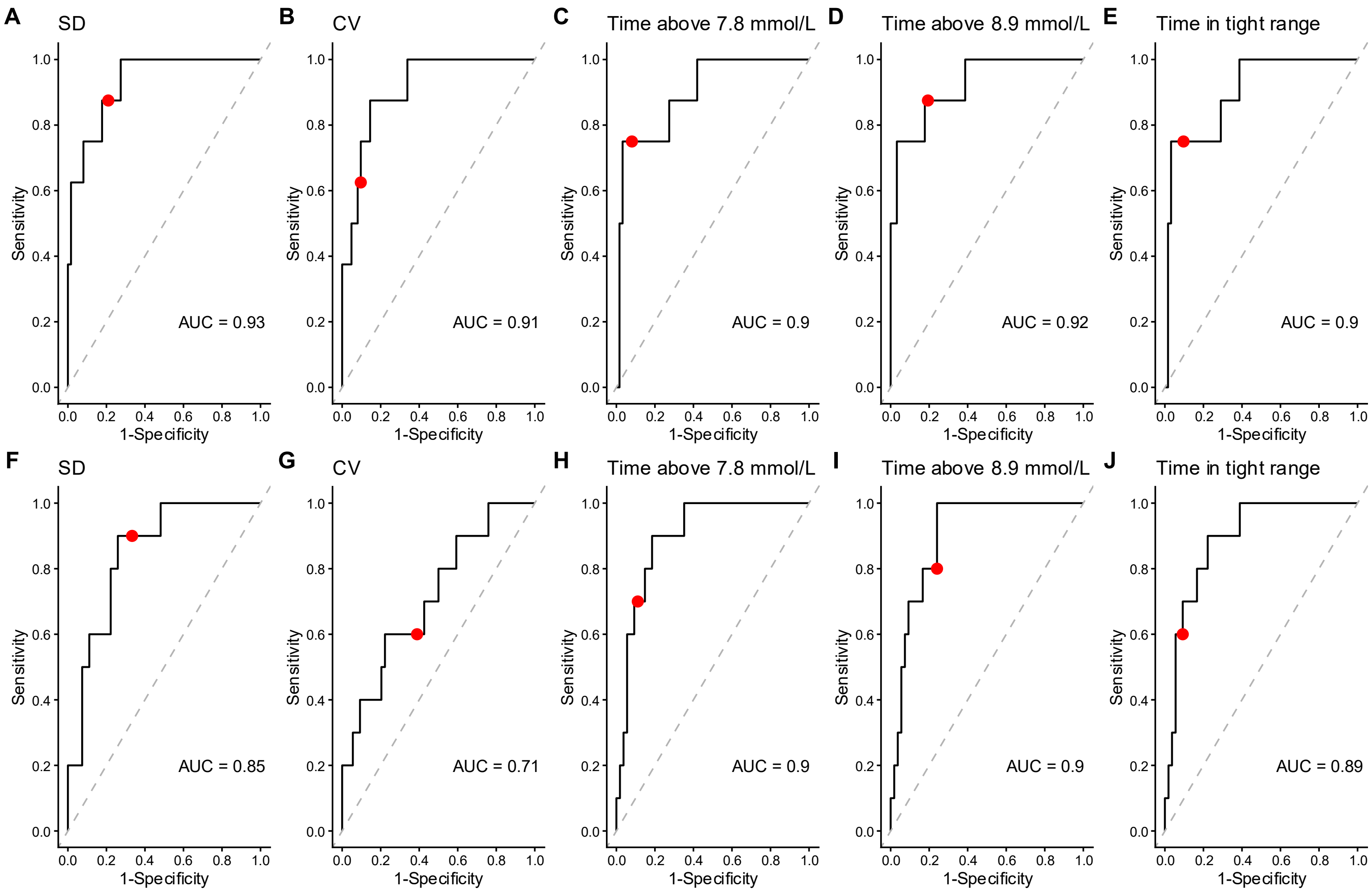

ESM Fig. 7. Receiver Operator Characteristic (ROC) curves for one year progression. Curves are shown for the indicated CGM parameters in the FreeStyle Libre Pro iQ cohort (A-E) and the Dexcom G6 cohort (F-J). Participants were categorized into those who progressed to stage 3 type 1 diabetes within 1 year of their CGM period (Sensitivity; FreeStyle Libre, n = 8; Dexcom G6, n = 10) and those who had been followed for at least one year without progressing to stage 3 type 1 diabetes (1 - Specificity; FreeStyle Libre, n = 50; Dexcom G6, n = 52). The red filled circle represents the values corresponding to the CGM parameter 97.5th centile of controls

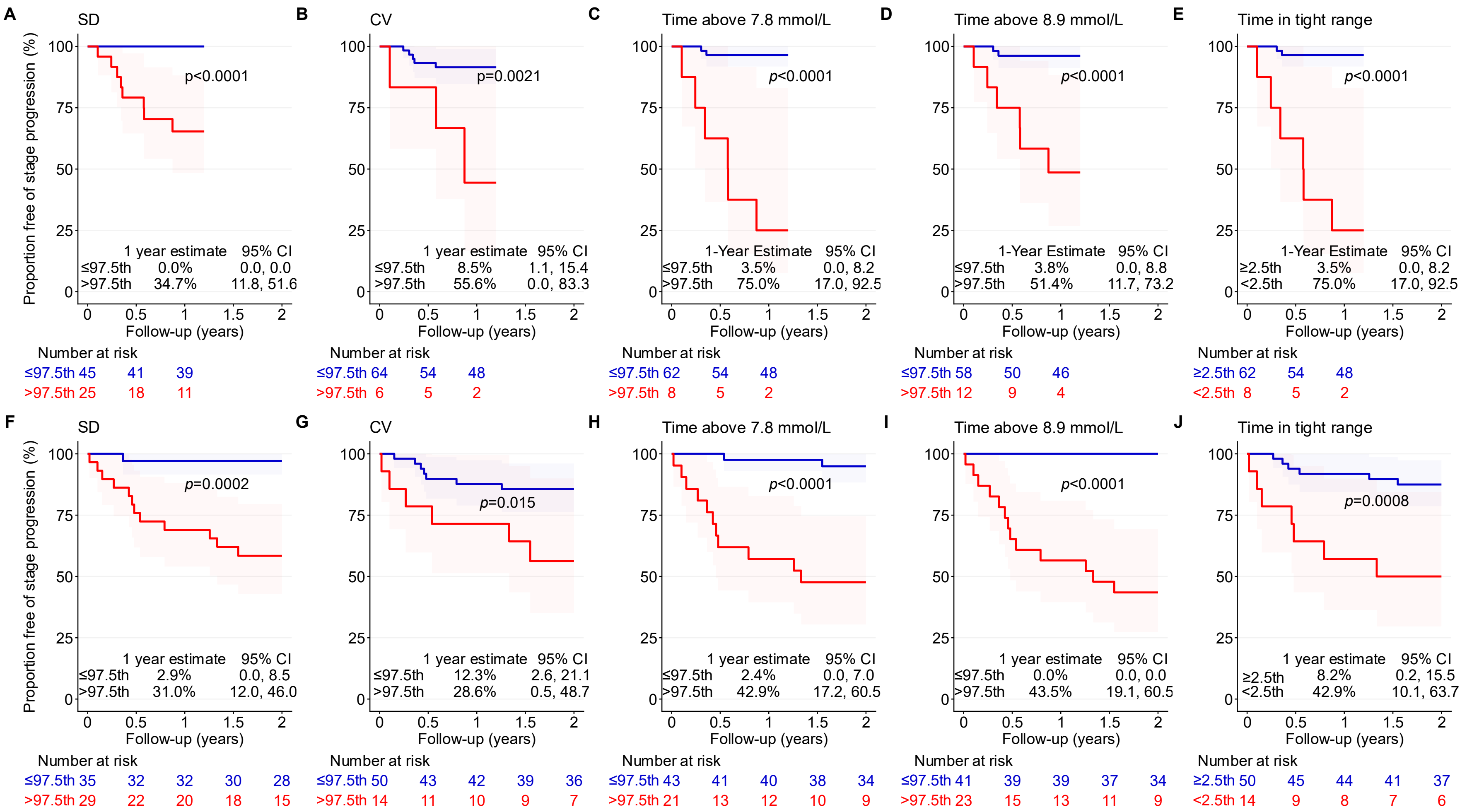

ESM Fig. 8. CGM parameter stratification of progression rate to stage 3 type 1 diabetes. Kaplan-Meier analysis of the proportion remaining without stage 3 type 1 diabetes after categorization of participants in the FreeStyle Libre Pro iQ cohort (A-E) and in the Dexcom G6 cohort (F-J) as above (red lines) or below (blue lines) the 97.5th percentile of harmonized values of control cohorts for standard deviation (A,F), coefficient of variation (B,G), time above 7.8 mmol/l (TA140) (C,H), time above 8.9 mmol/l (TA160) (D,I), and above and below the 2.5th percentile for time in tight range (E,J). The thresholds correspond to 1.1 mmol/l for SD, 23% for CV, 20% for TA140, 5% for TA160, and 75% for TITR
